# Intrasinusoidal ALK-positive anaplastic large cell lymphoma

**DOI:** 10.1007/s12308-025-00672-4

**Published:** 2025-12-10

**Authors:** Eilena Braye, Laurence de Leval

**Affiliations:** 1 Promed Laboratoire SA, Department of Pathology, Marly, Switzerland; 2 Institute of Pathology, Department of Laboratory Medicine and Pathology, Lausanne University Hospital and Lausanne University, Lausanne, Switzerland

**Keywords:** Anaplastic large cell lymphoma, ALCL, ALK positiv ALCL, T cell lymphoma

## Abstract

ALK-positive anaplastic large cell lymphoma (ALCL) may show sinusoidal involvement, but exclusive intrasinusoidal infiltration of lymph nodes is exceptionally rare. We describe a 39-year-old woman with isolated stage I inguinal lymphadenopathy, in whom the lymph node architecture was preserved despite massive intrasinusoidal infiltration by CD30- and ALK-positive atypical cells. This pattern can closely mimic metastatic carcinoma, creating a significant diagnostic challenge. Its recognition is crucial, as ALK-positive ALCL generally responds well to therapy. Further studies are required to better define the clinical and prognostic implications of this presentation.

A 39-year-old woman presented with isolated left inguinal lymphadenopathy. One excised lymph node showed an overall preserved architecture with florid follicular hyperplasia and paracortical expansion. In addition, the sinusoids in the subcapsular, cortical, and medullary regions were markedly distended by large atypical cells forming solid aggregates (Fig. 1A, D, G). By immunohistochemistry (Fig. 1B–C, E–F, H–L), the cells were strongly positive for CD30 and ALK (cytoplasmic and nuclear), with expression of CD2 (±), CD4 (dim), CD43 (-/+), TIA-1, Granzyme B, EMA, and MUM1. CD20 and other T cell markers (CD3, CD5, CD7, CD8) were negative. In situ hybridization for EBV (EBER) was negative. Ki-67 index exceeded 95%. Endothelial markers highlighted the vascular framework surrounding the neoplastic cells. A diagnosis of ALK-positive anaplastic large cell lymphoma (ALCL) was rendered. PET scan confirmed localized stage I disease.

**Fig. 1:**
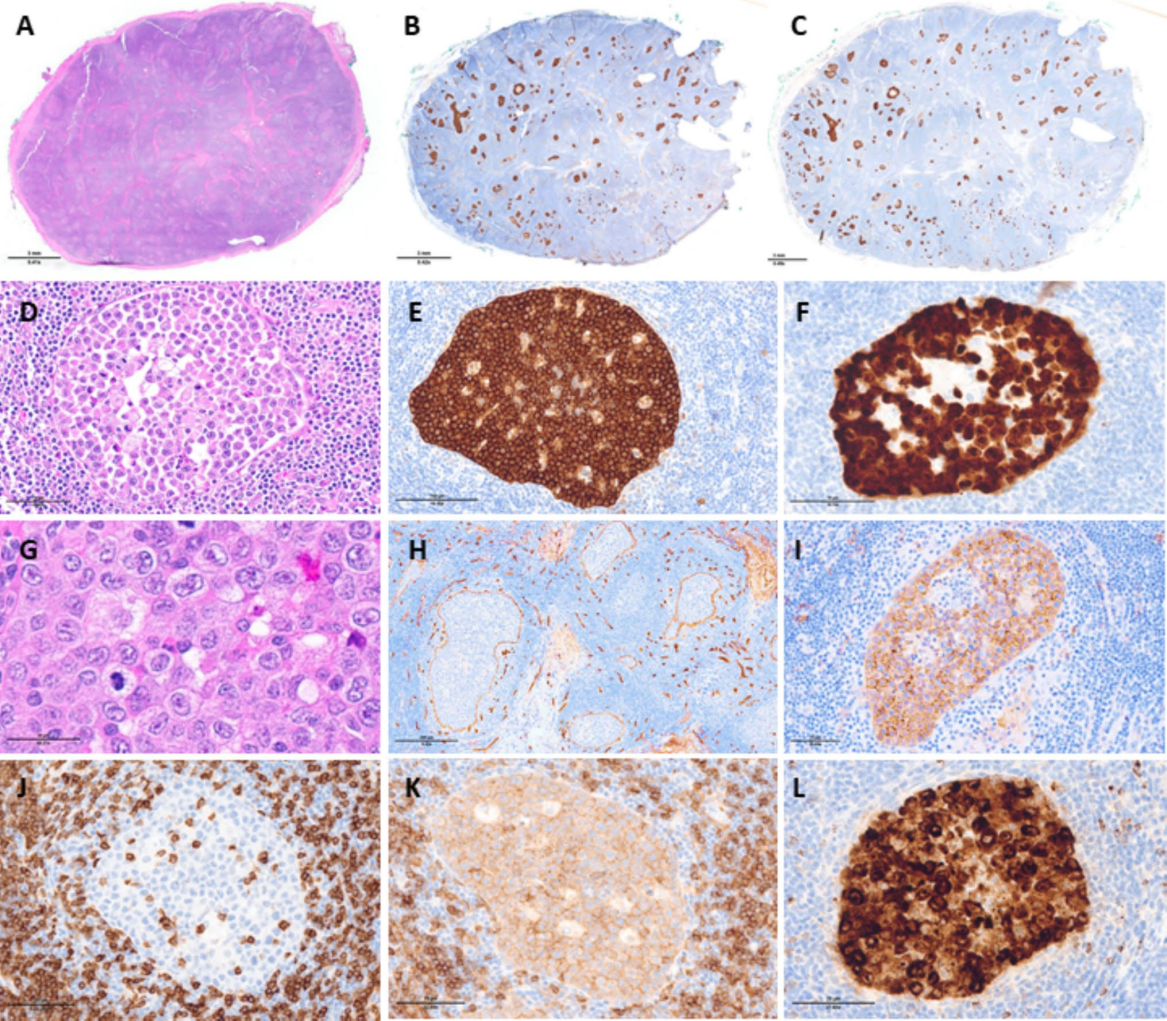
Intrasinusoidal ALK-positive anaplastic large cell lymphoma. **A** Panoramic view of the left inguinal lymph node biopsy, showing preserved nodal architecture with follicular hyperplasia and paracortical expansion (H&E, ×20). **B**, **C** Panoramic view of immunostains for CD30 (**B**) and ALK (**C**) highlighting multiple tumor cell aggregates molding the sinusoidal lumens (immunoperoxidase, ×20). **D** Focus on a sinusoid distended by atypical cells (H&E, ×200). **E** Strong and diffuse CD30 positivity in the large atypical cells (immunoperoxidase, ×200). **F** Nuclear and cytoplasmic positivity for ALK in neoplastic cells (immunoperoxidase, ×200). **G** High magnification of the neoplastic cells having abundant eosinophilic to pale cytoplasm and oval to indented nuclei with variably prominent nucleoli, including hallmark cells (H&E,×400). **H** CD34 expression in endothelial cells and highlightingsinusoidal pattern (immunoperoxidase, ×100); **I**–**J** Immunostains demonstrating expression of EMA (**I**), lack of CD3 (**J**), dim staining for CD4 (**K**), and intense positivity for granzyme B (immunoperoxidase, ×200)

It is 40 years since ALCL was first recognized by H. Stein and colleagues, who described a series of lymphomas composed of large anaplastic cells often with abundant cytoplasm, frequent sinusoidal invasion, and positivity with Ki-1 antibody reflecting strong CD30 expression [1]. These tumors had been previously often misdiagnosed as other neoplasms, including malignant histiocytosis and metastatic carcinoma. Initially, no distinction was made between T cell or null versus B cell phenotypes, but in the subsequent classifications, ALCL was restricted to cases of T cell lineage, and systemic cases were further divided into ALK-positive and ALK-negative [2, 3].

Propensity for sinusoidal infiltration is typical of ALCL; however, purely intrasinusoidal involvement manifestation, as illustrated in this case, is exceptional and may be diagnostically challenging. The clinical correlates or potentially prognostic implications of this peculiar topographical pattern are unknown. Awareness of this pattern is essential to avoid misdiagnosis and ensure appropriate therapy.

## Data Availability

No datasets were generated or analysed during the current study.
